# Is Pasture Cropping a Valid Weed Management Tool?

**DOI:** 10.3390/plants9020135

**Published:** 2020-01-21

**Authors:** Ignacio M. Luna, César Fernández-Quintanilla, José Dorado

**Affiliations:** 1 Instituto Nacional de Tecnología Agropecuaria (INTA)-Estación Experimental Agropecuaria Quimilí, Ruta Prov N° 6 km 9, Santiago del Estero, Quimilí 3740, Argentina; 2 Instituto de Ciencias Agrarias (CSIC), Serrano 115B, 28006 Madrid, Spain

**Keywords:** Intercropping, winter barley, *Cynodon dactylon*, *Eragrostis curvula*, no-tillage, weeds

## Abstract

The aim of the present work was to study the feasibility of pasture cropping under the Mediterranean conditions prevailing in central Spain and its potential as a weed management tool. Three cropping systems were assessed: conventionally grown winter barley and winter barley in pasture cropping with two perennial summer species, *Cynodon dactylon* and *Eragrostis curvula*. The results showed that the growth of these two species in a pasture cropping system was limited by the severe drought conditions and high temperatures present during the summer in some of the study years. Although there were no differences in the establishment of winter barley in any of the treatments assessed, pasture cropping reduced winter barley yields up to 50%–60% in years with low rainfall in spring. Regarding weed control, pasture cropping showed a significant suppression of the total weed density and number of weed species. As a conclusion, pasture cropping can be considered as a valid weed management tool. However, the economic feasibility of this system under the climatic conditions of central Spain (characterized by a high risk of severe summer droughts) is still not clear. The availability of supplemental irrigation may reduce competition between pastures and winter crops and ensure a profitable production of summer pastures.

## 1. Introduction

Pasture cropping is a no-tillage technique consisting of sowing annual crops into living perennial pastures during their dormant stage [1,2]. This cropping system combines species with complementary growth periods to diversify the farming systems and improve overall land productivity. Potential environmental benefits of this system are varied, and include increasing soil cover, reducing erosive processes, improving soil structure and organic matter, increasing infiltration and water retention, reducing N lixiviation and sequestering CO_2_ [1,3,4]. 

Pasture cropping has been widely promoted in southern Australia, and the term has been used to describe any form of no-till seeding of cereal crops into pastures [5]. However, the most commonly accepted practice involves establishing summer active perennial pastures that are grazed up to the autumn, when winter cereals are directly drilled in the dormant sod [1,4,6]. In the spring, as temperatures increase, the pasture resumes growth when the winter cereal is in its final growth stage. However, since the cereal may enter grain filling, the yields of both cereal crops and pastures could be negatively affected [7]. Considering all these factors, it is critical to select pasture species that are able to grow under severe water stress during the Mediterranean summer and stay dormant during the winter period, resuming growth when the cereal has completed its vegetative and reproductive growth.

In previous studies, various C_4_ grasses have been used as pasture species under Australian conditions [1,4]. These species enter dormancy early in the autumn and stay in that state until late spring. In addition, they are well adapted to grow under water stress conditions, extracting water from deep soil horizons and making efficient use of this limited resource [8,9]. Several of these C_4_ grasses have being tested under Italian conditions with promising results [10]. In the climate conditions of central Spain, the introduction of perennial summer pastures into dominant cereal systems may be a promising but hazardous practice. As far as we know, no previous studies have been conducted in this regard.

Barley is one of the most important crops in Europe [11] and is generally considered very competitive against weeds [12]. Despite this, the barley crop has been widely cited as requiring weed management because of the potential yield losses caused by different weed species in commercial fields [13,14,15,16,17]. One of the potential benefits of pasture cropping is improved weed control. The weed suppression benefits of using cover crops has been reported in numerous research studies [18,19]. Cover crops can compete with weeds for resources such as light, water and nutrients [20]. In addition, they may release allelopathic substances that reduce weed density [21]. However, most previous studies have been conducted with winter cover crops and summer cash crops. According to various authors [4,22], summer-growing perennials have the potential to assist with controlling weeds. However, no experimental evidence is available to support this statement.

The aim of the present work was to study, under the Mediterranean conditions of central Spain, (a) the performance of various C_4_ perennial grasses in a pasture cropping system, (b) the response of winter barley to the presence of competing perennial grasses, and (c) the effects of this system on the weed community.

## 2. Results

### 2.1. Summer Pastures

Two similar studies were conducted from 2015 to 2017 (Study 1) and from 2017 to 2019 (Study 2). In Study 1, biomass production of *Cynodon dactylon* (L.) Pers. and *Eragrostis curvula* (Schrader) Nees. during the winter and spring (up to barley harvest in June) ranged between 1038 and 1719 kg dry matter per hectare, with no significant differences between the two species (Table 1). No data were recorded for *Panicum maximum* Jacq. and *Brachiaria* spp. (*B. ruziziensis × B. decumbens × B. brizantha*), since these two species were not able to survive the winter temperatures in central Spain (see Materials and Methods). Similarly, the 2018 data from Study 2 showed no differences between *C. dactylon* and *E. curvula*, with average production levels of about 2000 kg dry matter per hectare. However, the data from 2019, characterized by low rainfall in spring, showed an 87% decrease in *C. dactylon* and a 93% reduction in *E. curvula* biomass, compared to the previous year.

Biomass production during the summer and early autumn was assessed in November 2016 for Study 1, after early autumn rains and before the browning of the pastures. The results showed no significant differences between pasture species, with yields ranging between 2446 and 2704 kg dry matter per hectare. In Study 2, summer pasture production in 2018 was practically null due to a lack of summer and autumn rains. In contrast, summer biomass yields in 2019 were 4463 and 6209 kg dry matter per hectare for *C. dactylon* and *E. curvula*, respectively, as a result of abundant rainfall (191 mm) in the June–October period.

**Figure 1:**
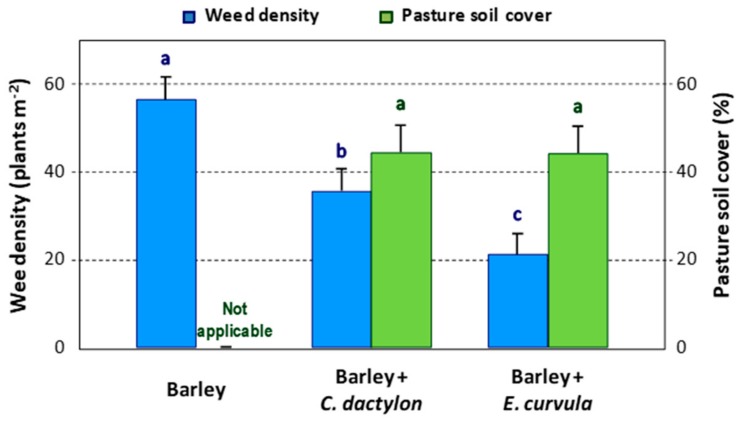
Weed density and pasture soil cover in the three treatments in 2019 in Study 2.

In Study 2, it was remarkable that pasture soil cover in intercropping plots decreased substantially from 2018 to 2019, reaching values close to 45% of the total plot area in both pasture species (Figure 1).

### 2.2. Winter Barley 

Barley establishment was not affected by pasture cropping in either of the two studies (Table 2). In Study 2, the barley density was lower in 2019 than in 2018, which was probably due to unfavorable environmental conditions in that year.

**Figure 2:**
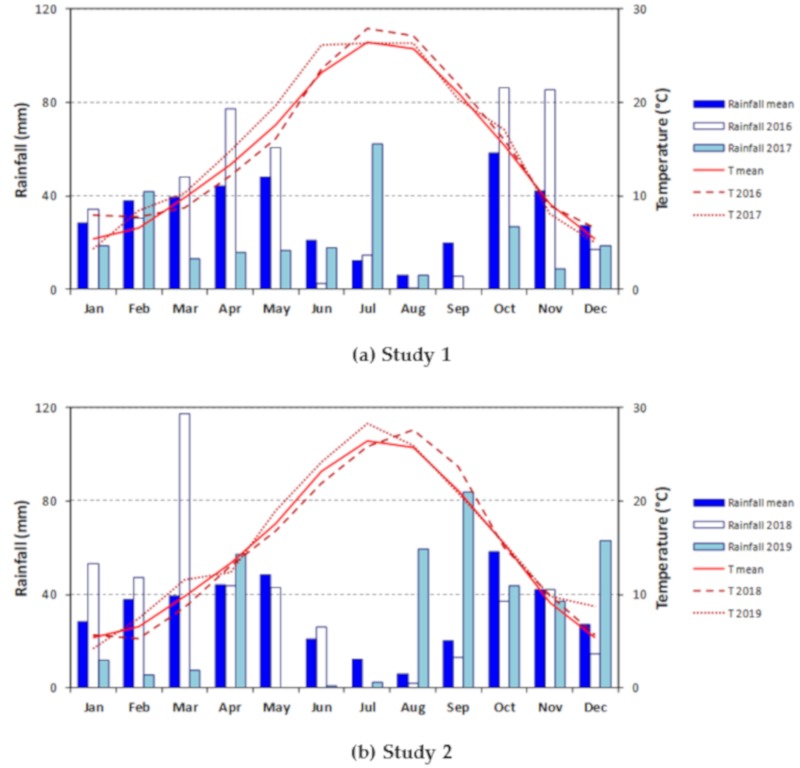
Monthly rainfall (mm) and temperature (°C), as well as the average of the 15-year series (2004–2018) for Arganda del Rey for (**a**) Study 1, 2016 and 2017; (**b**) Study 2, 2018 and 2019.

No significant differences between conventional cropping and intercropping were observed for barley yield when spring rainfall was above-average (2016 in Study 1 and 2018 in Study 2; Figure 2). In contrast, in years with below-average rainfall (2017 in Study 1 and 2019 in Study 2), crop yield differences were observed between treatments. In 2017, a higher barley yield was achieved in plots intercropped with *C dactylon*, apparently due to a better grain filling (Table 3). In 2019, the yields of the whole experiment decreased drastically due to very scarce rainfall in the months of greatest demand for the crop (March–May). This reduction was particularly severe in plots intercropped with *C. dactylon* and *E. curvula*, in which production was reduced by 50% and 61%, respectively. Apparently, this was due to the significantly lower number of spikes in these treatments (Table 3).

### 2.3. Weed Community

Total weed density was reduced as a result of the pasture cropping system in both studies (Table 4). The highest weed densities were observed in conventional barley compared to the two intercropping treatments. In Study 1, reductions of the total number of weed plants ranged between 80% and 86% for *E. curvula* and *C. dactylon*, respectively. Similarly, in Study 2, the reduction in the total number of weeds ranged from 80% (in plots with *C. dactylon*) to 90% (in plots with *E. curvula*) when pasture cropping was used.

The number of weed species was also significantly higher in conventional barley, with twice as many species than in pasture cropping in both studies (Table 4). The two dominant species, *Polygonum aviculare* L. in Study 1 and *Papaver* spp. in Study 2, were much more abundant in conventional barley than in the two pasture cropping treatments. Similarly, the control of other species such as *Descurainia sophia* (L.) Webb ex Prantl and *Lolium rigidum* (Gaudin) Weiss ex Nyman was favored by pasture cropping, mainly with *E. curvula*. In addition, other species that were negatively affected by pasture cropping were *Chenopodium album* L. (mainly in *C. dactylon*) in Study 1 and *Amsinckia calycina* (Moris) Chater in Study 2.

As previously indicated in Section 2.1, a reduction in the pasture soil cover by perennial pastures was found after two years of growing in Study 2. Despite both perennial species suffering a similar decrease of about 45% in pasture soil cover (Figure 1), the response of weeds was different, with *E. curvula* producing a significantly higher decrease in total weed density than *C. dactylon* (Table 4), thus showing a greater competitive ability against weeds.

## 3. Discussion

### 3.1. Summer Pastures

The performance of pasture cropping in Mediterranean climates depends on the ability of the perennial species to persist during prolonged periods of summer drought and to survive harsh winters by staying asleep or having very low growth rates [4]. In the specific conditions of central Spain, only two species (*E. curvula* or *C. dactylon*) of the four C_4_ grasses tested were able to survive winter conditions. *Eragrostis curvula* had already been shown to be well-adapted to the conditions of southern Italy [10]. *Cynodon dactylon* is widely distributed in most of Spain and is an important weed in several perennial crops [23]. The good adaptation and weedy behavior of *C. dactylon* represents a hazard for the artificial introduction of this species in pasture cropping systems. The establishment of summer-growing pastures under Mediterranean conditions may be another critical stage [4,10]. In our studies, irrigation was used to support pasture establishment in the first year. However, this could be a limiting factor in rainfed farms.

Pasture biomass production during the winter and early spring was mainly dependent on seasonal rainfall. In Study 2, the higher winter/spring biomass production in 2018 was linked to higher rainfall during this period (358 mm, see Materials and Methods). It is likely that, under these conditions, water was not an important limiting factor in the competition for resources between winter crop and summer pastures. In contrast, the low winter/spring biomass production in 2019 was linked to the scarce rainfall during the cereal growing season (140 mm, see Materials and Methods) and the likely competition for water between barley and pastures. These results are in accordance with previous work on pasture cropping systems [1,2,4].

Similarly, the summer biomass production of perennial pastures was very dependent on seasonal rainfall. Indeed, as cited in previous work with gatton panic (*Panicum maximum* cv. Gatton) in pasture cropping systems [1,4], production increased as the rainfall increased, with values ranging from 2000 kg ha^−1^ when rainfall was less than 150 mm during summer–autumn, to values of 3500 kg ha^−1^ [1] or even 7000 kg ha^−1^ [4]—the latter with accumulated rainfall close to 460 mm. In our research, we found comparable results, with lower productions in Study 1 corresponding to summer rainfall below 150 mm, and yields of over 6000 kg ha^−1^ in *E. curvula* in Study 2 in 2019, when an above-average rain of 191 mm occurred. These results confirm that, under Mediterranean conditions, with practically dry summers, late summer rainy events when temperatures are still favorable for pasture growth would be the most effective for the generation of pasture biomass, since spring rainfall would be used by the main cereal crop.

In addition to the limiting factor due to drought and high summer temperatures in Mediterranean conditions for pasture production, in this study, we found that the area occupied by pasture has decreased over time (about 45% in both species in the second year), despite the excellent establishment of both species with pasture soil covers close to 100% by first year. Consequently, the lower the pasture soil cover, the greater the area that weeds can occupy and increase their density progressively, which will limit the competitive capacity of perennial grasses over weeds—one of the main incentives for the use of pasture cropping. Among the causes that could explain the loss of pasture soil cover would be the need for water during the summer in order to synthesize photo-assimilates that will serve as an energy reserve. As Nie et al. [9] mention, the climate—and, in particular, rainfall—is an important factor affecting pasture persistence. These authors pointed out that, in regions with low summer rainfall, there appeared to be a trade-off between summer activity and persistence for temperate grasses, so that summer-dormant plants were more persistent than summer-active species. The 150 mm threshold has been cited as the minimum rainfall for pasture growth in Mediterranean climatic conditions when the intercropping system was used in Australia [4]. In our case, summer and early autumn rainfall in 2018 was below this amount, and that probably negatively affected the perennial pastures’ capacity to maintain pasture soil cover in 2019. Studies carried out in Italy [10] successfully implanted and maintained *E. curvula* for 3 years in different environments, although variable support irrigations were required during the summer. Indeed, when feasible, the practice of supplemental irrigation during the summer can be an interesting and useful tool to increase both the persistence and biomass production of pastures in Mediterranean climates.

### 3.2. Winter Barley 

The results of this study have confirmed, under Spanish conditions, the possibility of sowing winter cereals on perennial summer pastures with no effect on the establishment of winter barley. Pasture cropping has been already reported, under Australian conditions, as a useful way to maintain soil cover and reduce the consequences of cereal establishment failures in lighter soils characterized by low fertility and low soil water-holding capacity [22]. Specific meteorological conditions may require the use of specific practices. In our study, as a result of the warm, rainy autumn which occurred in 2018, pastures were still active at the time of barley planting, requiring herbicide treatment for pasture desiccation. This type of treatment has also been used in other cases [1].

The economic profitability of an intercropping system would be conditioned by the coexistence of both cereal crop and pasture without competition penalizing the crop yields [22]. Our results have shown that pasture cropping would strongly affect the main crop yield when the available resources, especially water, are low. Barley yield losses observed in a dry year (50% to 60% in 2019) are similar to those reported for wheat under pasture cropping [2,24] and are considerably higher than those reported on barley by Lawes et al. [1] and Thomas et al. [25], at 26% and 12%, respectively. The lower impact on barley yield in these latter experiences could be due to lower pasture growth during cereal senescence in late spring, relative to our observations. In fact, the 2019 results in our study were conditioned by low rainfall, thus causing a high degree of competition for resources between perennial grasses and winter barley at that time. In general, we found the regrowth of the pasture during mid-April (data not shown), coinciding with the cereal phenological stage of spike emerging when water demand is highest. The greater availability of water in 2018 would be enough to supply cereal and pasture needs, with no statistical differences between conventional and intercropping systems, while in 2019, the lack of water was a limiting factor. Previous work studying soil water dynamics in a pasture cropping system in southwestern Australia [7] indicated that perennial pastures reduced soil water content during the growing period of both species by 150 mm compared to conventional management, clearly demonstrating the competition for water between the pasture and cereal crop. In addition, the authors of this study pointed out that the water use efficiency of grain production was significantly higher for barley in conventional than in pasture cropping, suggesting that the difference was driven by both lower yield in the pasture cropping treatments and higher water use efficiency. 

### 3.3. Weed Community

Our results showed that, regardless of environmental conditions, pasture cropping had a significant suppression effect on the total weed density and number of weed species. This suppressive effect could be related to early competition for light as a consequence of pasture soil coverage. In this regard, in [26], which studied the effect of different cover crops on weed performance, the authors concluded that autumn weed suppression was positively correlated to light interception by the cover crop. Apparently, early interception is more likely to condition competition than late light interception. In pasture cropping, a similar effect could be assumed due to the abundant residues of perennial grasses maintained in the soil in early stages of winter cereal. Similarly, in crop systems involving non-tillage (i.e., maintaining crop residues on the soil surface), a decrease in weed infestation has been reported, which was directly related to the percentage of residues in the soil [27].

Since barley was directly drilled into the pasture, this limited soil disturbance is likely to have similar effects on the weed community than those reported in no-tillage studies [28,29]. The suppression of *P. aviculare* and *C. album* observed in our study under pasture cropping was already reported for no-tillage systems in other studies [28,30,31]. In contrast, different results have been found for other species. In our study, the densities of *L. rigidum*, *P. rhoeas* and *D. sophia* were reduced in the two pasture systems, whereas these same species were favored by no-tillage in other studies [32,33,34]. These results would indicate that these species, with small-sized seeds which are well adapted to germinate at or near the surface [35], could suffer some interference with pastures (either competition or allelopathy) that would explain a decrease in density with respect to conventional tillage.

The presence of allelopathic substances generated by perennial pastures could be hypothesized in processes of competition between pastures and weeds. Indeed, the allelopathic potential of *E. curvula* has been cited in previous studies carried out by Ghebrehiwot et al. [36]. These authors concluded that *E. curvula* had a greater allelopathic potential than the rest of the species tested, thus partly explaining the success of this species in South African grasslands. A related species, *Eragrostis plana,* has also been cited for allelopathic effects from various plant parts [37,38]. These researchers attributed an important role to the residues of this species maintained on the soil surface. They suggested that the allelochemicals would be released during the decomposition of these residues in such a way that the seeds with a rapid germination escaped the allelopathic effect of *E. plana*, while seeds that delayed germination were exposed to the allelopathic effects [38]. This could explain the null effect of perennial pastures on barley in our studies. Barley seeds have a rapid germination and establishment after sowing, while weed seeds with a longer germination pattern in time could be affected by allelochemicals released from perennial pastures. On the other hand, *C. dactylon* has also been documented for its allelopathic effects on other plant species, both from aqueous extracts of its rhizomes [39] and from extracts of stems [40].

Consequently, pasture cropping could assist in controlling weeds in no-till systems, avoiding the exclusive dependence on the use of glyphosate. This product is the world’s most widely used herbicide and it is currently essential in non-plough tillage systems. However, its use has been highly questioned in recent years, to the extent that its approval for use in Europe after 15 December 2022 is pending [41]. In addition, the intercropping system with direct-drilled winter cereals would be an efficient management of weeds in organic farming, avoiding ploughing and therefore improving the structure and biological health of the soils.

## 4. Materials and Methods 

### 4.1. Site and Meteorological Data

Two field studies were conducted from 2015 to 2017 (Study 1) and from 2017 to 2019 (Study 2) at “La Poveda” research farm in Arganda del Rey, Madrid (Lat. 40°18′49.4″ N Long. 3°29′15.3″ W). This farm is flat, and its soil type is classified as a Xerofluvent, sandy loam textural class (United States Department of Agriculture (USDA) classification) in the first half meter, with a progressive increase in the proportion of sand up to 1.5 m in depth, at which gravel begins to appear. The site has a semiarid Mediterranean climate with a dry and hot summer period, and the mean annual temperature and rainfall (over the last 15 years) in this area were 14.9 °C and 385 mm, respectively, with a cereal growing season (November–June) average rainfall of 289 mm. Over the course of Study 1 (explained further below), the 2016 and 2017 annual and barley growing season (parentheses) rainfall amounts for Arganda del Rey were 462 (280) and 247 (227) mm. Despite the fact that annual rainfall was considerably lower in 2017, the precipitations during the barley growing season were similar. In Study 2 (explained further below), 2018 and 2019 annual and growing season (parentheses) rainfall amounts for Arganda del Rey were 440 (358) and 373 (140) mm. This represented 24% more rainfall during the winter crop growing season for the first year and 50% less in the second. The long-term mean and monthly rainfall graphics are presented in Figure 2. 

Assuming a period of summer growth of pastures from June to October, the average rainfall for this period is 118 mm, while the rainfall recorded in this season was 109 mm in 2016 and 113 mm in 2017 for Study 1, as well as 78 mm in 2018 and 191 mm in 2019 for Study 2 (Figure 2).

### 4.2. Experimental Design and General Management

#### 4.2.1. Study 1

Originally, a randomized complete block design (RCBD) with four replications and five treatments were established in 3 m × 15 m plots. The treatments included winter barley (*Hordeum vulgare* L. cv Hispanic) grown as a monoculture and four pasture cropping treatments with barley drilled on four C_4_ perennial grasses (*C. dactylon*, *E. curvula*, *P. maximum* and *Brachiaria* spp.). Perennial grass species were selected for their promising results under Australian [1,4,7], Italian [10] and North American [42,43] conditions. However, although all of them were successfully established during the first summer and autumn, *P. maximum* and *Brachiaria* spp. were not able to survive the first winter (despite temperatures not being below the average of the last 15 years in the study area). Consequently, in this study, we finally had only three treatments: barley, barley + *C. dactylon* and barley + *E. curvula*. The four C_4_ grasses were hand-seeded in May 2015 at rates of 10–20 kg ha^−1^ (depending on the species). During the first summer, the experiment was sprinkle-irrigated to ensure grass establishment. Barley was sown in late November in 2015 and 2016, after the grasses had entered into winter dormancy, at a rate of 180 kg ha^−1^. In the conventional barley treatment, this crop was seeded with a conventional planter after conventional tillage (one pass of the disc harrow and two passes of the rotocultivator). In the two pasture cropping treatments, barley was directly drilled after glyphosate (3.0 L ha^−1^ Roundup 36%) treatment. Fertilizers were applied prior to barley seeding (350 kg ha^−1^ of a 8N–11P_2_O_5_–11K_2_O complex) and at late tillering (192 kg ha^−1^ of Ammonium Nitrosulphate 26%) in all treatments. No post-emergence herbicides were applied in any treatment.

#### 4.2.2. Study 2

The experimental design was the same as in the previous study, using an RCBD with four replications and three treatments: conventional barley and two pasture cropping treatments—*C. dactylon* and *E. curvula* intercropped with barley. The plot size was increased (10 m × 20 m) in order to increase the sampling intensity. The two pastures were hand-sown in April 2017 at rates of 8 and 10 kg ha^−1^ for *C. dactylon* and *E. curvula*, respectively, supporting their establishment with irrigation up to September. Barley (cv. Hispanic) was sown in late November in 2017 and early December in 2018, using similar agronomic practices as in the previous study. During the first cropping cycle, no herbicides were applied to any of the three treatments. In the second cycle, after the autumn mowing of perennial grasses, carfentrazone (0.3 L ha^−1^ Carfentrazone Etil 22.3%) was applied to both pasture species on 27 November 2018 before barley sowing to dry the green material. This desiccant treatment was necessary because of the lack of winter dormancy caused by unseasonable warm temperatures. Post-emergence herbicides (pinoxadem 42 g a.i. ha^−1^ and tifensulfuron-methyl 50% + tribenuron-methyl 25% 30 g a.i. ha^−1^) were applied on 28 February 2019 only to conventional barley.

### 4.3. Perennial Pastures, Barley Crop and Weeds Measurements

The pasture biomass was monitored two times: in June (just before barley harvest), to quantify spring production, and in November (prior to barley planting), to quantify summer and early autumn production. Spring biomass was assessed during the four years considered in the two studies. Summer biomass was only measured in 2016 (Study 1), a year with an average rainfall, and in 2019 (Study 2), a year characterized by above-average rainfall. Aboveground pastures were mowed in three 0.25 m^2^ quadrats per plot in late June (2016 to 2019) before barley harvest and November (2016 and 2019) before barley sowing, then dried in an oven at 105 °C for 24 h to determine the dry weight yield. Visual assessments of pasture soil cover were conducted on photographic images. A total of 15 digital images per plot were obtained in a regularly spaced 3 m × 4 m grid at the end of June 2019, once the barley was harvested. Pasture soil cover in each image was assessed independently by three experienced observers using a methodology similar to that explained by Andújar et al. [44].

The density of the winter barley was monitored in January–February, depending on the year, before crop tillering by counting the barley seedlings in ten 0.11 m^2^ quadrats in each plot. The barley grain yield was measured in June, harvesting manually four 0.25 m^2^ quadrats in each plot. Other crop yield parameters such as the number of spikes and the 1000-grain weight were also counted in the same samples collected in these 0.25 m^2^ quadrats.

Weed density was assessed out at the end of winter (March 2016 to 2018, and February 2019) just before post-emergence herbicide treatment in conventional barley (if applied) by counting the number of seedlings of each weed species in six 0.11 m^2^ quadrats in each plot, then calculating the number of species in each sample.

### 4.4. Statical Analisys

Data related to perennial pasture (e.g., biomass yield) and winter barley (crop density, number of spikes, 1000-grain weight and grain yield) were assessed independently for each year in both experiments (Study 1: 2016 and 2017; Study 2: 2018 and 2019), using a linear mixed-effects model (lme procedure; nlme library from R statistical software) to analyze an RCBD, with management (conventional vs. pasture cropping) as a fixed effect and replication as a random effect.

Weed data (number of species, weed density) were analyzed separately in each study using a generalized linear mixed model (glmer procedure; lme4 library from R statistical software) in an RCBD, with management in the model as a fixed effect and year and replication as random effects.

Heteroscedasticity was modeled when necessary using the function of variance identity (varIdent, R statistical software). Akaike Information Criteria and Bayesian Information Criteria were used to determine the best model for each variable. Then, inferences were made about the means of the treatments with the Least Significant Difference (LSD) test with a significance level of α = 0.05. All analyses were performed with the InfoStat statistical software and its communication interface with R software [45].

## 5. Conclusions

Regarding the question of whether pasture cropping is a valid weed management tool, according to the results of this study, the answer is yes, since this technique showed a significant suppression of the total weed density and number of weed species. Nevertheless, pasture cropping appears not to be well adapted to the environmental conditions of central Spain—at least in the manner in which it was originally proposed. Indeed, yield losses of the main crop due to pasture cropping were high in low-rainfall scenarios because perennial grasses began to regenerate at the same time as the winter crop entered the reproductive stages. Under no irrigation management, this condition limits the sustainability of the whole system, compromising the productivity of the main crop. In addition, in rainfed farming, summer pastures only provide sporadic and unpredictable feed for livestock according to summer rainfall, apart from the fact that pasture soil cover is likely to decrease over time as a result of low rainfall, which would affect the weed control effect. Supplemental irrigation—the addition of limited amounts of water to essentially rainfed crops to stabilize yields during dry spells—may be an effective response to alleviating these adverse effects. In this regard, future work should aim to investigate the effects of irrigation management to avoid or reduce yield losses due to competition between pastures and winter crop.

## Figures and Tables

**Table 1:** *Cynodon dactylon* and *Eragrostis curvula* biomass (kg dry matter ha^−1^) under pasture cropping management.

	Study 1			Study 2		
	(kg Dry Matter ha^−1^ ± SE *)			(kg Dry Matter ha^−1^ ± SE *)		
	Spring production		Summer production	Spring production		Summer production
	2016	2017	2016	2018	2019	2019
*C. dactylon*	1719 ± 359 a	1146 ± 379 a	2446 ± 618 a	2041 ± 373 a	142 ± 60 a	4463 ± 118 b
*E. curvula*	1038 ± 359 a	1192 ± 379 a	2704 ± 623 a	2000 ± 307 a	265 ± 60 a	6209 ± 471 a

* SE = standard error. Means in a column within an experiment and year followed by the same letter are not significantly different (*P* > 0.05) according to Least Significant Difference (LSD) Fisher.

**Table 2:** Barley density (plants m^−2^) in the three treatments in Studies 1 and 2.

	Study 1 (Plants m^−2^ ± SE *)		Study 2 (Plants m^−2^ ± SE)	
	2016	2017	2018	2019
Barley	255 ± 14 a	230 ± 7 a	268 ± 9 a	203 ± 7 a
Barley + *C. dactylon*	261 ± 14 a	216 ± 5 a	257 ± 8 a	182 ± 7 a
Barley + *E. curvula*	289 ± 13 a	238 ± 8 a	278 ± 9 a	188 ± 7 a

* SE = standard error. Means in a column within an experiment and year followed by the same letter are not significantly different (*P* > 0.05) according to LSD Fisher.

**Table 3:** Barley yield parameters (number of spikes, grain weight per spike and grain yield) in the three treatments in Studies 1 and 2.

	**Study 1**					
	Number of Spikes (N° m^−2^ ± SE *)		1000-Grain Weight (g ± SE)		Grain Yield (kg ha^−1^ ± SE)	
	2016	2017	2016	2017	2016	2017
Barley	493 ± 41 a	379 ± 28 a	39.3 ± 2.3 a	36.1 ± 2.0 b	4238 ± 311 a	1893 ± 166 b
Barley + *C. dactylon*	542 ± 45 a	460 ± 33 a	44.3 ± 2.3 a	42.2 ± 2.0 a	4091 ± 311 a	2518 ± 166 a
Barley + *E. curvula*	499 ± 42 a	375 ± 27 a	45.4 ± 2.3 a	37.3 ± 2.0 ab	3699 ± 311 a	1846 ± 166 b
	**Study 2**					
	Number of Spikes (N° m^−2^ ± SE *)		1000-Grain Weight (g ± SE)		Grain Yield (kg ha^−1^ ± SE)	
	2018	2019	2018	2019	2018	2019
Barley	577 ± 48 a	359 ± 27 a	46.7 ± 1.1 a	25.8 ± 2.5 a	4810 ± 541 a	1020 ± 85 a
Barley + *C. dactylon*	572 ± 47 a	243 ± 19 b	44.7 ± 1.1 a	30.0 ± 2.7 a	4081 ± 541 a	514 ± 85 b
Barley + *E. curvula*	597 ± 49 a	193 ± 15 c	46.8 ± 1.1 a	18.8 ± 2.5 b	4245 ± 541 a	401 ± 85 c

* SE = standard error. Means in a column within an experiment and year followed by the same letter are not significantly different (*P* > 0.05) according to LSD Fisher.

**Table 4:** Weed establishment and species (Number and density) in the three treatments in Studies 1 and 2.

	**Study 1**		
	Barley	Barley + *C. dactylon*	Barley + *E. curvula*
N^o^ species m^−2^	4.72 ± 0.36 a	2.07 ± 0.27 b	2.43 ± 0.29 b
Weed density (plants m^−2^)*			
Total species	322.88 ± 145.61 a	45.70 ± 20.79 b	64.94 ± 29.48 b
*Chenopodium album*	9.17 ± 6.27 a	0.96 ± 0.77 b	1.93 ± 1.51 ab
*Descurainia sophia*	0.10 ± 0.22 a	0.03 ± 0.07 b	0.00 ± 0.00 c
*Lolium rigidum*	0.07 ± 0.22 a	0.03 ± 0.11 b	0.03 ± 0.11 b
*Papaver* spp.	41.10 ± 13.91 a	18.42 ± 6.60 b	38.89 ± 14.91 a
*Polygonum aviculare*	214.15 ± 131.75 a	14.23 ± 8.58 b	12.93 ± 7.79 b
	**Study 2**		
	Barley	Barley + *C. dactylon*	Barley + *E. curvula*
N^o^ species m^−2^	2.56 ± 0.49 a	1.38 ± 0.30 b	0.95 ± 0.22 b
Weed density (plants m^−2^)*			
Total species	117.69 ± 44.38 a	23.84 ± 9.30 b	11.72 ± 4.60 c
*Amsinckia calycina*	0.42 ± 0.88 a	0.01 ± 0.03 b	0.01 ± 0.02 b
*Descurainia sophia*	4.44 ± 2.24 a	3.63 ± 2.17 ab	0.82 ± 0.49 b
*Lolium rigidum*	9.70 ± 2.15 a	6.56 ± 2.34 ab	3.32 ± 1.85 b
*Papaver* spp.	83.11 ± 61.87 a	3.33 ± 2.55 b	4.86 ± 3.70 b
*Polygonum aviculare*	4.18 ± 6.00 a	0.08 ± 0.13 b	0.15 ± 0.23 b

Means ± standard error in a row within an experiment followed by the same letter are not significantly different (*P* > 0.05) according to LSD Fisher. * Only species with statically significance are shown.
